# Measurement and Visualization of Tight Rock Exposed to CO_2_ Using NMR Relaxometry and MRI

**DOI:** 10.1038/srep44354

**Published:** 2017-03-10

**Authors:** Haitao Wang, Zengmin Lun, Chengyuan Lv, Dongjiang Lang, Bingyu Ji, Ming Luo, Weiyi Pan, Rui Wang, Kai Gong

**Affiliations:** 1 State Key Laboratory of Shale Oil and Gas Enrichment Mechanisms and Effective Development, Beijing, 100083, China; 2 Petroleum Exploration & Production Research Institute (PEPRIS), Sinopec, Beijing, 100083, China

## Abstract

Understanding mechanisms of oil mobilization of tight matrix during CO_2_ injection is crucial for CO_2_ enhanced oil recovery (EOR) and sequestration engineering design. In this study exposure behavior between CO_2_ and tight rock of the Ordos Basin has been studied experimentally by using nuclear magnetic resonance transverse relaxation time (NMR T_2_) spectrum and magnetic resonance imaging (MRI) under the reservoir pressure and temperature. Quantitative analysis of recovery at the pore scale and visualization of oil mobilization are achieved. Effects of CO_2_ injection, exposure times and pressure on recovery performance have been investigated. The experimental results indicate that oil in all pores can be gradually mobilized to the surface of rock by CO_2_ injection. Oil mobilization in tight rock is time-consuming while oil on the surface of tight rock can be mobilized easily. CO_2_ injection can effectively mobilize oil in all pores of tight rock, especially big size pores. This understanding of process of matrix exposed to CO_2_ could support the CO_2_ EOR in tight reservoirs.

Tight oil is a new and unconventional resource. According to the U.S. Energy Information Administration (EIA) data, as of 2013 tight oil is more than 42% of U.S. domestic total crude oil production and it is expected to reach 59% in 2020. In China, tight oil has been explored in five main basins^1^,^2^, especially, Yanchang Formation of the Ordos Basin that started commercial production in 2012^3^. The reservoirs in CHANG 8 layer of the Ordos Basin are tight sandstones with average matrix permeability of 0.3 mD^4^. Horizontal well fracturing has been carried out, but production rapidly decreased. Recently, CO_2_ enhanced oil recovery (EOR) pilot has been planned in this oilfield. CO_2_ EOR is a process in which CO_2_ under supercritical conditions acts as a powerful solvent and is routinely used for extracting more oil out of aging reservoirs^5^. Meanwhile, CO_2_ EOR also considerably reduces greenhouse gas emissions^6^. The commonly recognized CO_2_ EOR mechanisms include the oil viscosity reduction, oil swelling effect, interfacial tension (IFT) reduction, light-hydrocarbons extraction in immiscible and miscible conditions^7^,^8^,^9^. However, the injected CO_2_ will flow along fractures and cannot permeate into rock matrix due to low matrix permeability in tight oil reservoir. So it is of significant importance to study the mechanisms of oil mobilization in CO_2_ injection process in tight reservoir.

Recently, a large numbers of studies about CO_2_ EOR in tight reservoir have been conducted. Hawthorne *et al*.^10^ performed the exposure experiments between CO_2_ Bakken tight rock and proposed conceptual mechanisms of CO_2_ EOR in a tight fractured system: (1) CO_2_ flows into and through the fractures, (2) rock matrix is exposed to CO_2_, (3) CO_2_ permeates the rock under pressure, then swelling of crude oil extrudes some oil out of the pores, (4) oil migrates to the bulk CO_2_ via swelling and reduced viscosity, and (5) oil production is slowly driven by concentration gradient diffusion from pores into the bulk CO_2_ in the fractures. Zekri *et al*.^11^ reported that immiscible CO_2_ was capable of mobilizing oil in the rock with very low permeability (0.16 mD) and providing reasonable displacement efficiency. Furthermore, immiscible CO_2_ recovery was probably related to ability of oil extraction by supercritical CO_2_^12^. Shyeh-Yung^13^ concluded that two mechanisms of CO_2_ EOR were low interfacial tension (IFT) displacement and extraction of oil components, and the latter dominated after CO_2_ breakthrough. Vega, *et al*.^14^ conducted the miscible injection of CO_2_ into siliceous shale. More than 54% of oil can be recovered by CO_2_ injection. CO_2_ diffusion was a major recovery mechanism, which results in a decrease in oil viscosity and an increase in CO_2_ molar fraction in the oil. Tovar *et al*.^15^ evaluated CO_2_ EOR in unconventional liquid reservoirs (ULR) with nanodarcy matrix permeability. CO_2_ was able to penetrate the cores, resulting in an estimated oil recovery in the range of 18–55%. Oil vaporization into the carbon dioxide was the main mechanism for oil production. In conclusion, CO_2_ diffusion into matrix to mobilize oil is the main mechanism of CO_2_ EOR in the tight matrix.

NMR technology, including NMR T_2_ spectrum and MRI, is a quantitative and visual analytical tool^16^. The absolute pore radius distribution can be obtained with NMR T_2_ distribution calibrated by mercury injection capillary pressure (MICP). Recently, NMR T_2_ distribution was conducted to characterize the tight reservoir. Saidian and Prasad^17^ measured the NMR T_2_ distributions of Middle Bakken formation. Wang *et al*.^18^ determined the water distribution of tight sandstones (0.109–0.336 mD) by NMR T_2_ distributions. Marschall *et al*.^19^ conducted the NMR T_2_ test of rock samples covering a wide range of permeability (0.078–4450 mD).

There have been many studies on the visualization of flow and transport in porous media using MRI techniques. Zhao *et al*.^20^ visualized the process of CO_2_ being injected into the bead-pack core (grain size distribution ranging from 0.177–0.250 mm) at high pressure and high temperature by using MRI. The piston-like miscible regions and CO_2_ front, onset of CO_2_ channeling or fingering, and the distribution of oil in porous media can be accurately detected. Suekane *et al*.^21^,^22^ used MRI technique to directly image the distribution of supercritical CO_2_ injected into a packed bed of glass beads (with 70 um average diameter) and Berea sandstone (permeability of 19.6 mD and porosity of 18.5%.) containing water. Water saturation distributions can be successfully observed. Brautaset *et al*.^23^ investigated the fluid saturation distributions and monitored the fluid flow characteristics *in situ* by MRI during water flooding followed by CO_2_ in four Portland Chalk core samples with different wettability.

Understanding mechanisms of oil mobilization of tight matrix during CO_2_ injection is crucial for CO_2_ EOR engineering design in fractured tight oil reservoir. Oil mobilization characteristic of tight matrix in pore scale during CO_2_ injection is the key scientific question for effective production of fractured tight oil reservoir. In this study, we focused on the process of tight matrix exposed to CO_2_ under the temperature of 40 °C and pressure of 12 MPa and 22 MPa. NMR T_2_ and MRI were used to detect the tight matrix exposure process *in situ* to study mechanisms of oil mobilization during CO_2_ injection. After tight matrix was exposed to CO_2_, NMR T_2_ test and MRI were continuously performed until the obtained T_2_ spectrum remained unchanged to investigate the effect of exposure time on oil mobilization. At the end of the first exposure experiment, interaction between CO_2_ and tight matrix reached equilibrium state. Then the second exposure experiment started with CO_2_ injection under a constant pressure of 12 MPa and at a constant rate to displace the CO_2_ used in the first exposure experiment in order to remain concentration of CO_2_ to be 100% in the gas phase. CO_2_ injection process also was detected by NMR to study oil mobilization in the CO_2_ displacement process. Finally, 2^nd^, 3^rd^ and 4^th^ exposure experiments were conducted to investigate the effect of exposure times sequentially. Effect of immiscible and miscible pressure on oil mobilization was also investigated.

## Results

### NMR T_2_ spectra of CO_2_ exposure experiment

**Figure 1:**
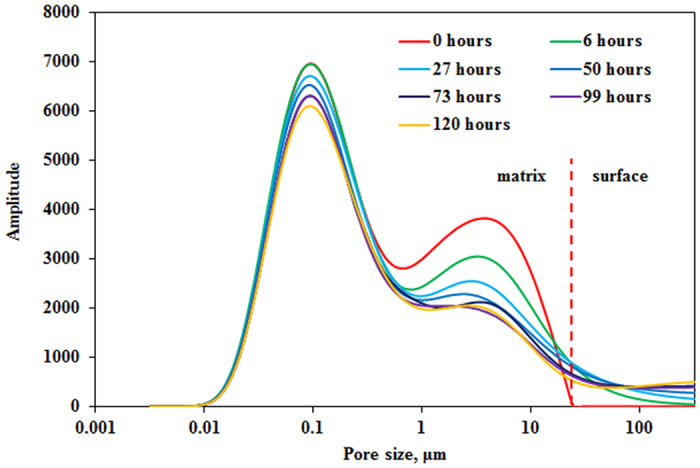
NMR T_2_ spectra of exposure experiment under different exposure time.

Exposure experiment was conducted between CO_2_ and matrix with permeability of 0.2180 mD at 40 °C and 12 MPa. Tight matrix was exposed to CO_2_ and T_2_ test of tight matrix was continuously performed with NMR system. NMR T_2_ spectra of CO_2_ exposure experiment under different exposure time are shown in Fig. 1. It can be observed that oil in all pores can be mobilized as exposure time increases. Note that NMR T_2_ amplitude reduction of pores with radius larger than 1 μm is greater than that of pores with radius smaller than 1 μm. Therefore, the recovery of oil in the former is larger. Initially, oil exists in pores with maximum radius of 21 μm in the original saturated tight matrix. After CO_2_ injection, oil flows to pores with radius greater than 21 μm, suggesting that oil in the tight matrix “diffuses” to the surface of matrix. And NMR T_2_ amplitude of pore with radius greater than 21 μm increases as exposure time increases, which indicates that more oil gradually “diffuses” to the surface of tight matrix. From NMR T_2_ amplitude reduction, the recovery of oil in pores with radius larger than 1 μm increases more sharply in initial exposure stage. For example, NMR T_2_ amplitude reduction of pores with radius larger than 1 μm from 0 to 27 hours is greater than that from 27 hours to 50 hours. Similar phenomenon was also observed in pores with radius smaller than 1 μm, but the amplitude of NMR T_2_ reduction is relatively small.

### Recoveries and MRI of CO_2_ exposure experiment

**Figure 2:**
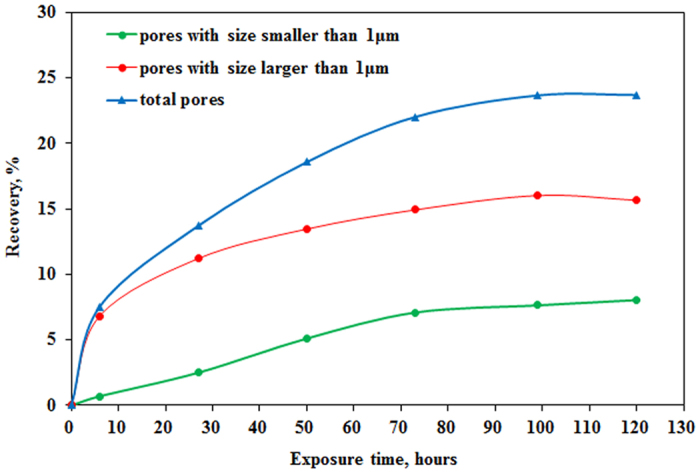
Recoveries of oil in different pore size versus exposure time.

**Figure 3:**
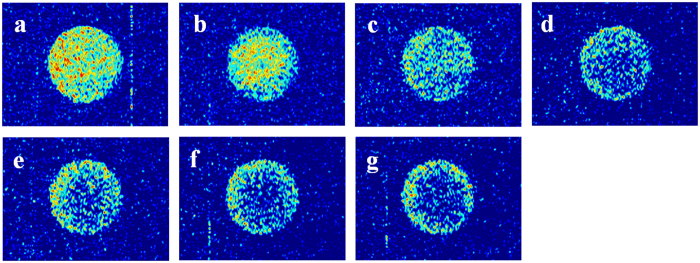
MRI images of core in the first exposure experiment: (**a**) 0 h; (**b**) 6 h; (**c**) 27 h; (**d**) 49 h; (**e**) 73 h; (**f**) 99 h; (**g**) 120 h.

Recoveries of oil in different pore size and MRI of tight matrix versus exposure time are shown in Figs 2 and 3, respectively. Two stages can be divided according to the recovery curve: a fast-growing stage (the recovery increases up to 13.7% within initial 27 h) and a slow-growing stage (the recovery increases from 13.7% to 23.7% during the next 93 h). During the fast-growing stage (0–27 h) MRI images of tight core show that oil can be mobilized homogenously along the perimeter of core and oil saturation of core decreases from the outside inward (Fig. 3b). After 27 h oil saturation (Fig. 3c) becomes homogeneous and is lower than that of the initial condition (Fig. 3a), which means CO_2_ has diffused into the whole core. During the slow-growing stage (27–120 h) oil saturation of core decreases from the inside outward from MRI images (Fig. 3d–g). The oil saturation of core shows slower variation and recovery increases less in the slow-growing stage compared with the fast-growing stage. The recovery is 15.7% for oil in pores with radius larger than 1 μm, which is higher than the recovery of 8.0% for oil in pores with radius smaller than 1 μm (Fig. 2).

### NMR T_2_ spectra of CO_2_ injection process

**Figure 4:**
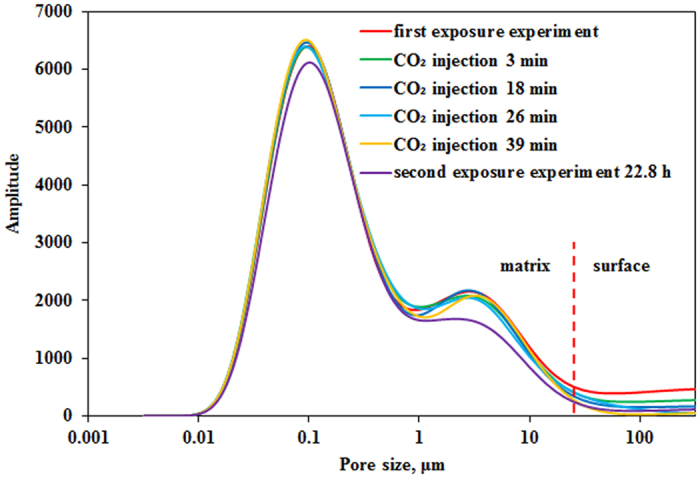
NMR T_2_ spectra of CO_2_ injection process and second exposure experiment.

**Figure 5:**
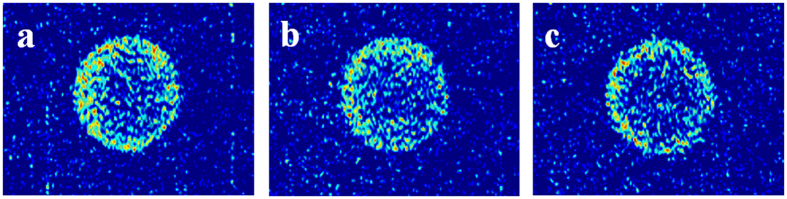
MRI images of core in the second exposure experiment: (**a**) 1 h; (**b**) 5 h; (**c**) 23 h.

After the first exposure experiment, interaction between CO_2_ and tight matrix reached equilibrium state. Then the second exposure experiment started with CO_2_ injection under a constant pressure of 12 MPa and at a constant rate to remain CO_2_ fresh in system. The same procedure of NMR T_2_ test was followed during CO_2_ injection and second exposure experiment. Figure 4 shows NMR T_2_ spectra of CO_2_ injection process and second exposure experiment. The NMR T_2_ amplitude of pores with radius greater than 21 μm decreases as CO_2_ injection time increases, which indicates that oil on the surface of tight matrix is displaced by CO_2_ injection. However, NMR T_2_ amplitude of pores with radius smaller than 21 μm almost remains unchanged, suggesting that oil in the tight matrix cannot be mobilized during CO_2_ injection process. CO_2_ injection stopped and second exposure experiment started after 39 min. Note that oil in the tight matrix can also be mobilized after second exposure for 22.8 h. MRI images of tight core in the second exposure experiment are shown in Fig. 5. Oil saturation gradual reduction of core can be observed from the inside outward.

### Exposure times

**Figure 6:**
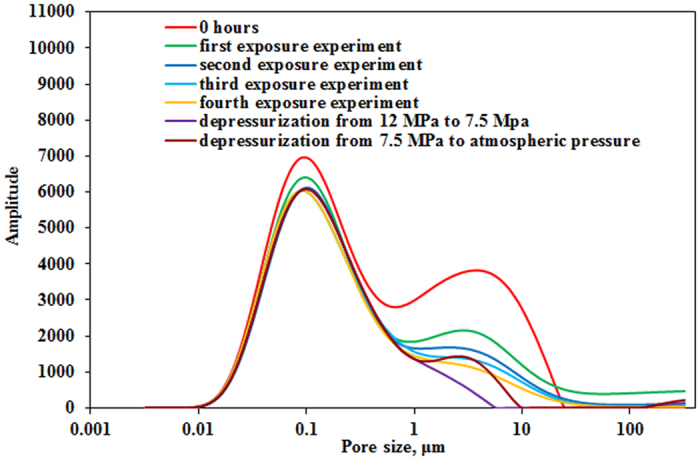
NMR T_2_ spectra at the end of 1^st^, 2^nd^, 3^rd^ and 4^th^ exposure experiments and two depressurization experiments.

**Figure 7:**
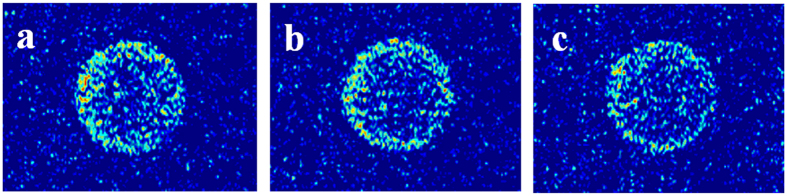
MRI images of core in the third exposure experiment: (**a**) 1 h; (**b**) 5 h; (**c**) 23 h.

NMR T_2_ spectra at the end of 1^st^, 2^nd^, 3^rd^ and 4^th^ exposure experiments are presented in Fig. 6. It can be observed that NMR T_2_ amplitude of tight matrix gradually decreases at the end of 1^st^, 2^nd^, 3^rd^ and 4^th^ exposure experiments, which indicates that oil in the tight matrix can be gradually mobilized as exposure times increases. Note that oil mobilization in pores with radius larger than 1 μm is more significant than that in pores with radius smaller than 1 μm. Especially at the end of 3^rd^ and 4^th^ exposure experiments, oil in pores with radius smaller than 1 μm remains nearly unchanged. MRI images of tight core in the 3^rd^ exposure experiment indicate that oil saturation shows slight variation (Fig. 7). A small portion of residual oil can be observed (Fig. 7c). After 4^th^ exposure experiment, depressurization experiment including two stages was conducted: in the first stage the pressure decreased from 12 MPa to 7.5 MPa (near critical pressure) and in the second stage pressure decreased from 7.5 MPa to atmospheric pressure. Figure 6 shows that oil in pores with radius larger than 1 μm can also be mobilized during the process of depressurization, EOR of partial big pores even reaches 100%.

**Figure 8:**
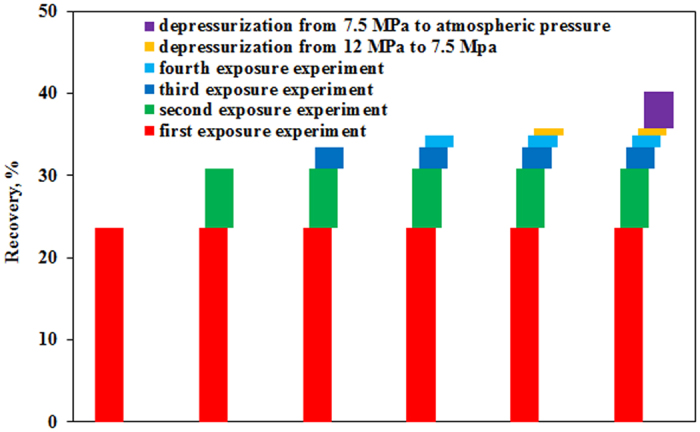
Final recoveries of 1^st^, 2^nd^, 3^rd^ and 4^th^ exposure experiments and two depressurization experiments.

Figure 8 shows the final recoveries of four exposure experiments and depressurization experiment. The final recoveries of 1^st^, 2^nd^, 3^rd^ and 4^th^ exposure experiments are 23.7%, 7.2%, 2.6% and 1.5%, respectively and decrease as exposure times increases. Recoveries of two depressurization experiments are 0.9% and 4.5%, respectively.

### Immiscible and miscible pressure

**Figure 9:**
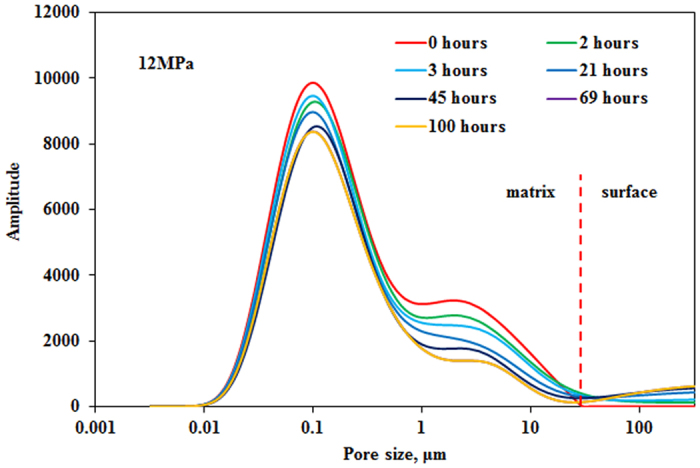
NMR T_2_ spectra of exposure experiment under immiscible pressure of 12 MPa.

**Figure 10:**
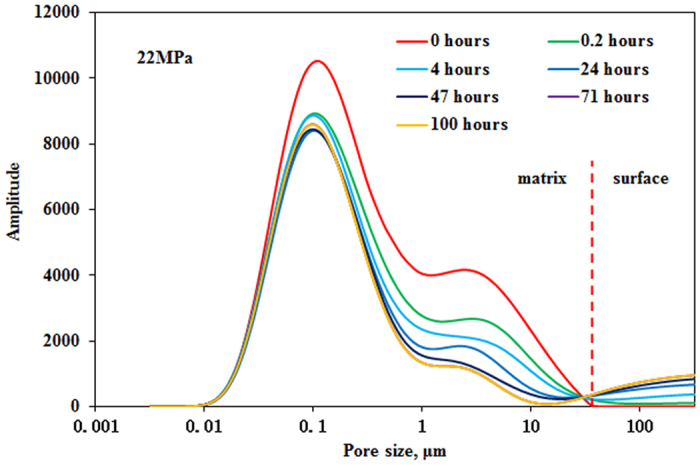
NMR T_2_ spectra of exposure experiment under miscible pressure of 22 MPa.

**Figure 11:**
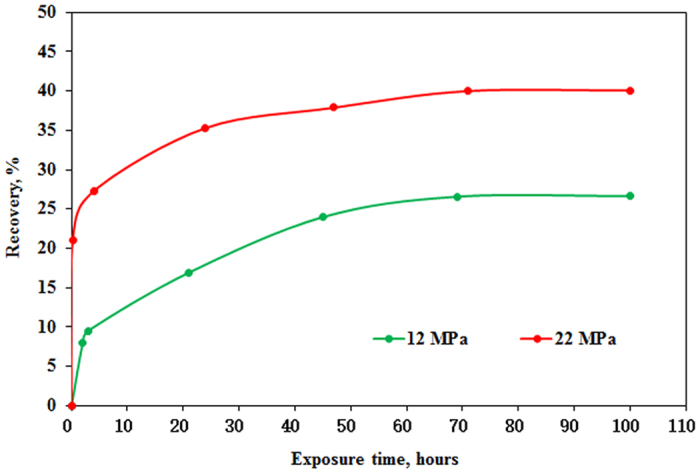
Recoveries of oil under different pressure versus exposure time.

Exposure experiments were conducted between CO_2_ and matrix under immiscible pressure of 12 MPa and miscible pressure of 22 MPa and temperature of 40 °C with core permeability of 0.5997 mD and 0.6038 mD, respectively. NMR T_2_ spectra of CO_2_ exposure experiment under different pressure are shown in Figs 9 and 10. As previously mentioned oil in all pores can be mobilized as exposure time increases. NMR T_2_ amplitude reduction of pores with radius larger than 1 μm is greater than that of pores with radius smaller than 1 μm. It can also be observed that oil in the tight matrix “diffuses” to the surface of matrix and more oil “diffuses” to the surface of tight matrix as exposure time increases. Recoveries of oil under different pressure versus exposure time are shown in Fig. 11. Two stages can be divided according to the recovery curve: a fast-growing stage and a slow-growing stage. It is obvious that recoveries of two exposure experiments increase sharply in the fast-growing stage, especially exposure experiment under miscible pressure of 22 MPa.

## Discussion

In the fast-growing stage of the first exposure experiment (0–27 h), CO_2_ diffuses into oil in the tight matrix, then oil swells and leaves from pores to surface. Oil signal can be observed on the surface of tight matrix (Fig. 1) and increases as exposure time increases, which indicates more oil swells to surface. MRI results (Fig. 3) demonstrate that CO_2_ diffuses into oil in the tight matrix. Main mechanism of oil mobilization is oil swelling due to CO_2_ diffusion into oil in the core. Oil concentration gradient diffusion also occurs while CO_2_ diffuses. Oil moves from pores with big size to pores with small size due to capillary force in oil-wet tight matrix, so the recovery of oil in pores with big size is higher than that of oil in pores with small size.

At the beginning of slow-growing stage of the first exposure experiment (27–120 h), CO_2_ has diffused into the whole tight matrix and nearly contacted all the oils, at the same time oil in all pores swells. Oil diffuses gradually from the inside outward and oil saturation of core decreases from MRI images from the inside outward (Fig. 3d–g). Main mechanism of oil mobilization is oil concentration gradient diffusion and capillary force, rather than oil swelling. Obviously oil concentration gradient diffusion is very slow process, which results in a slow increase of recovery.

In 2^nd^, 3^rd^ and 4^th^ exposure experiments, interaction between CO_2_ and tight matrix has reached equilibrium and oil swelling no longer exists. MRI results (Fig. 5) show that oil diffuses out the tight core and into CO_2_ phase. Oil mobilization in pores with radius larger than 1 μm is more significant than that in pores with radius smaller than 1 μm (Fig. 6). Especially at the end of 3^rd^ and 4^th^ exposure experiments, oil in pores with radius smaller than 1 μm remains nearly unchanged. This results can be attributed to that light components of oil have been extracted into CO_2_ phase in 1^st^ and 2^nd^ exposure experiments and residual heavy components, especially in small pore, cannot be mobilized in 3^rd^ and 4^th^ exposure experiments. Total recovery increases but amplitude decreases as exposure times increase. Recovery of the fourth exposure experiment is mere 1.5%.

The results of CO_2_ injection process (Fig. 4) indicate that oil in fracture can be easily mobilized but oil mobilization in the tight matrix just can be observed after 22.8 h in second exposure experiment and is time-consuming. So it is key to mobilize oil from tight matrix into fracture for fractured tight reservoir development. Meanwhile, a longer development time is required. Depressurization experiment process is similar to CO_2_ huff and puff, in which the main mechanism is dissolved gas displacement. And oil in pores with radius larger than 1 μm can also be mobilized in the process of depressurization (Fig. 6).

It can be observed that recovery in the fast-growing stage of exposure experiment increases sharply after pressure increases from 12 MPa to 22 MPa (Fig. 11). This result shows that CO_2_ contacts the oil faster and swells the oil more and extracts the light components more as pressure increases. However, pressure is higher than MMP of 17.8 MPa determined by slim tube test at 40 °C, but CO_2_ still can’t achieve miscibility with the oil (Figs 10 and 11). This result can be attributed to two reasons. First, pore and throat of tight rock are very small, so CO_2_ is difficult to contact the oil due to dynamic factor. Second, slim tube test to determine MMP is a multicontact miscibility process between CO_2_ and the oil. However, exposure experiment is a single contact process between CO_2_ and the oil. So MMP in exposure experiment is higher than that determined by slim tube test.

At the end of first exposure experiment CO_2_ utilization (PV of CO_2_ injected/PV of cumulative oil produced) of exposure experiment with permeability of 0.2180 mD under immiscible pressure of 12 MPa, exposure experiment with permeability of 0.5997 mD under immiscible pressure of 12 MPa and exposure experiment with permeability of 0.6038 mD under miscible pressure of 22 MPa is 64.3, 49.7 and 32.8, respectively. These CO_2_ utilizations are much less than that mentioned by Trivedi and Babadagli^24^. That is mainly attributed to that this study focuses on the oil mobilization on the condition of excess of CO_2_. Meanwhile, it can be observed that CO_2_ utilization increases from 64.3 to 49.7 as permeability of core increases from 0.2180 mD to 0.5997 mD under immiscible pressure of 12 MPa and even CO_2_ utilization can increase up to 32.8 after pressure raises to miscible pressure of 22 MPa.

Oil mobilization of tight matrix during CO_2_ EOR process is the most important for fractured tight oil reservoirs. The main oil recovery mechanism is diffusion in fractured reservoirs during injection of CO_2_ for EOR. Darvish *et al*.^25^ confirmed that diffusion was the main oil recovery mechanism through a variable produced oil composition in fractured reservoirs. Eide *et al*.^26^ demonstrated that diffusion was a viable oil recovery mechanism in fractured reservoirs and calculated an effective diffusion coefficient using dynamic 3D fluid saturations from computed-tomography (CT) with analytical methods. In this study oil mobilization was visualized and oil production in the pore scale was measurement during CO_2_ injection using NMR T_2_ spectrum and MRI. Injected CO_2_ diffused into oil in the tight matrix and then oil swelled and oil composition diffused. These effects could mobilize oil from tight matrix to fracture. Meanwhile, increasing pressure could quickly recover more oil from tight matrix. Minimum miscibility pressure (MMP) between oil and CO_2_ was 17.8 MPa determined by slim tube test at 40 °C. However, miscibility was not observed in this study under the miscible pressure of 22 MPa. This results indicated that MMP determined by traditional slim tube is not suitable and more influences should be considered for CO_2_ EOR design of fractured tight oil reservoirs.

## Methods

### Exposure experiment apparatus

Exposure experiment between CO_2_ and matrix was design for NMR measurement. The key units are NMR measurement system (PKU University) and exposure experiment device compatible for NMR test with a maximum temperature of 80 °C and a maximum pressure of 35 MPa. The NMR measurement system has a constant magnetic field strength of 2350 gauss (permanent magnets) and a resonance frequency of 10 MHz. The parameters for NMR T_2_ measurement were set as follows: echo time (TE), 0.23 ms; repetition time (TR), 2 s; echo numbers, 4096; numbers of scans, 64. After the measurements, transverse relaxation time (T_2_) was calculated by multi-exponential inversion of the echo data with 64 preset decay times logarithmically spaced from 0.1 ms to 10 s. The parameters for MRI were set as follows: echo spacing (TE), 3.2 ms; repetition time (TR), 500 ms; image data matrix, 256 × 256; field of view (FOV), 150 mm × 150 mm with a thickness of 20 mm.

### Experimental samples

Oil and tight matrix were collected from CHANG 8 layer of the Ordos Basin in Honghe Oilfield. Density and viscosity of oil were determined to be 0.8050 g/cm^3^ (at 40 °C) and 5.50 mPa.s (at 40 °C), respectively, at atmospheric pressure. Minimum miscibility pressure (MMP) between oil and CO_2_ was 17.8 MPa determined by slim tube test at 40 °C.

Horizontal cylindrical core plugs were cut and then were prepared for exposure experiment according to following procedures. 1. Cleaning core procedure. The core plug was held in a suitable core-holding device under overburden pressure that will permit the flow of solvent through the matrix. Toluene was injected from the inlet of core-holding device to rinse the oil for two weeks and then methanol was injected to rinse the brine for one week in the core. Then the rinsed core was dried for two days in the oven at 116 °C. An NMR T_2_ test of cleaned core clearly showed that there were no any signals of fluid inside the cleaned core. 2. Saturating and aging core procedure. The core was vacuumed up to 10^−5^ mbar for 48 h in the core-holding device under overburden pressure using molecular pump. Then the core was saturated with oil and aged at 80 °C and 30 MPa for 14 days. In this study effect of water saturation wasn’t investigated and so there was no procedure to establish the initial water saturation.

### Experimental procedures

The experimental procedure used in this study is briefly described as follows. The saturated core was enclosed into exposure experiment device. After the temperature of the whole system achieved setting experimental temperature and became stable, the core was exposed to CO_2_. The first exposure experiment started. Meanwhile, NMR T_2_ and MRI test of the core were continuously performed with the NMR system to investigate the effects of exposure time on EOR. At the end of the first exposure experiment, interaction between CO_2_ and core matrix reached equilibrium state with unchanged T_2_ spectrum. Then the second exposure experiment started with CO_2_ injection under a constant pressure of 12 MPa and at a constant rate to remain fresh CO_2_ in system. The procedure of NMR test was unchanged. The third and fourth exposures were conducted sequentially.

### Correlation of NMR T_2_ relaxation time and MICP pore throat radius

As for the NMR transverse relaxation time, T_2_, of a fluid in a pore is given by the following equation (1).


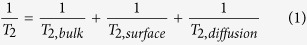


where T_2,bulk_ is the bulk relaxation time of the pore-filling fluid (ms), T_2,surface_ is the surface relaxation time (ms), and T_2,diffusion_ is the relaxation time as induced by diffusion (ms). As for fluid flow in porous media, T_2,bulk_ is usually neglected because T_2,bulk_ is much larger than T_2,surface_. T_2,diffusion_ is also neglected when the magnetic field used is deemed to be uniform with a quite small field gradient. Then T_2_ is mainly dependent on T_2,surface_, which is associated with specific surface area of a pore. T_2,surface_ can be expressed as the following equation (2).


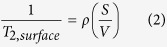


where ρ is the surface relaxivity (μm/ms), S is the pore surface area (μm^2^), and V is the pore volume (μm^3^). S/V can be rewritten as a function of the dimensionless shape factor of a pore, F_s_, and pore throat radius, r (μm) by the following equation (3).


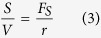


Combining equations (2) and (3), T_2, surface_ can be expressed as the following equation (4).


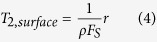


Surface relaxivity (ρ) and shape factor (F_s_) of a given core can be assumed to be constant. Thus, T_2_ can be rewritten as equation (5).


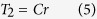


where C = 1/(ρF_s_), and C is a constant conversion coefficient (ms/μm).

According to Equation (4) 1/(ρF_s_) is introduced to account for the fact that NMR responds to pore body size whereas MICP is controlled by the size of pore throats. According to equation (5), relaxation time T_2_ can be converted into pore throat radius r using a constant conversion coefficient C. Thus conversion coefficient C that scales relaxation time T_2_ into MICP average pore throat radius r is determined by a method mentioned by Saidian and Prasad^12^. This method calculates the conversion coefficient C by combination of NMR time average (T_2LM_) (equation (6) and average pore throat radius measured by MICP, i.e. T_2LM_ = Cr_average_. T_2LM_ and average pore throat radius measured by MICP in this study are 13.26 ms and 0.427 μm, respectively. Conversion coefficient C is calculated to be 31.05 ms/μm.


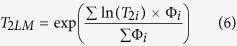


where T_2LM_ is NMR time average. Φ_i_ is the amplitudes of NMR T_2i_.

### Error analysis

Porosity, permeability, density and viscosity were measured five times, respectively. Accuracy of NMR test is analyzed based on signal of peak area in original saturated core. All results are shown in Table 1.

Arithmetic Mean (AM) is calculate using equation (7).


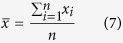


Relative average deviation (RAD) and relative standard deviation (RSD) in percentages are calculated using equations (8) and (9), respectively.


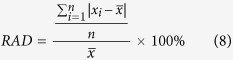



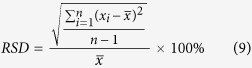


RAD and RSD of permeability test is less than 2% and RAD and RSD of porosity test is less than 1%. RAD and RSD of density test is less than 0.05% and RAD and RSD of viscosity test is less than 0.5%. RAD and RSD of NMR test is less than 0.6%.

## Additional Information

**How to cite this article:** Wang, H. *et al*. Measurement and Visualization of Tight Rock Exposed to CO_2_ Using NMR Relaxometry and MRI. *Sci. Rep.* **7**, 44354; doi: 10.1038/srep44354 (2017).

**Publisher's note:** Springer Nature remains neutral with regard to jurisdictional claims in published maps and institutional affiliations.

## Figures and Tables

**Table 1:** Test results and error analysis for routine core analysis, oil property and NMR.

Item	Test 1	Test 2	Test 3	Test 4	Test 5	AM	RAD (%)	RSD (%)
Permeability of Core 1 (mD)	0.2179	0.2131	0.2164	0.2211	0.2215	0.2180	1.21	1.60
Porosity of Core 1 (%)	9.5	9.5	9.4	9.6	9.5	9.5	0.42	0.74
Permeability of Core 2 (mD)	0.6022	0.6095	0.5988	0.5908	0.5911	0.5997	1.09	1.56
Porosity of Core 2 (%)	12.1	12.2	12.2	12.1	12.3	12.2	0.53	0.69
Permeability of Core 3 (mD)	0.6095	0.5988	0.6112	0.5963	0.5923	0.6038	1.23	1.75
Porosity of Core 3 (%)	12.3	12.4	12.2	12.3	12.5	12.3	0.71	0.92
Oil Density at 40 °C (g/cm^3^)	0.8049	0.8052	0.8050	0.8051	0.8048	0.8050	0.01	0.02
Oil Viscosity at 40 °C (mPa·s)	5.49	5.48	5.51	5.5	5.52	5.50	0.22	0.29
NMR Signal of Core 1 (Peak Area)	146016	146352	146517	147332	145980	145905	0.27	0.38
NMR Signal of Core 2 (Peak Area)	173188	173529	172647	171538	174107	173002	0.42	0.56
NMR Signal of Core 3 (Peak Area)	199488	199678	198269	199340	197295	198814	0.42	0.51
